# Cancer-associated fibroblasts predict poor outcome and promote periostin-dependent invasion in oesophageal adenocarcinoma

**DOI:** 10.1002/path.4467

**Published:** 2015-01-08

**Authors:** Timothy J Underwood, Annette L Hayden, Mathieu Derouet, Edwin Garcia, Fergus Noble, Michael J White, Steve Thirdborough, Abbie Mead, Nicholas Clemons, Massimiliano Mellone, Chudy Uzoho, John N Primrose, Jeremy P Blaydes, Gareth J Thomas

**Affiliations:** 1 Cancer Sciences Unit, Somers Cancer Research Building, University of SouthamptonUK; 2 University Health Network, Thoracic Surgery Clinic, University of TorontoCanada; 3 Division of Cancer Research, Peter MacCallum Cancer CentreEast Melbourne, Victoria, Australia; 4 Sir Peter MacCallum Department of Oncology, University of Melbourne, ParkvilleVictoria, Australia; 5 Department of Surgery (St. Vincent's Hospital), University of MelbourneParkville, Victoria, Australia

**Keywords:** CAFs, tumour microenvironment, oesophageal cancer, periostin

## Abstract

Interactions between cancer cells and cancer-associated fibroblasts (CAFs) play an important role in tumour development and progression. In this study we investigated the functional role of CAFs in oesophageal adenocarcinoma (EAC). We used immunochemistry to analyse a cohort of 183 EAC patients for CAF markers related to disease mortality. We characterized CAFs and normal oesophageal fibroblasts (NOFs) using western blotting, immunofluorescence and gel contraction. Transwell assays, 3D organotypic culture and xenograft models were used to examine the effects on EAC cell function and to dissect molecular mechanisms regulating invasion. Most EACs (93%) contained CAFs with a myofibroblastic (α-SMA-positive) phenotype, which correlated significantly with poor survival [*p =* 0.016; HR 7. 1 (1.7–29.4)]. Primary CAFs isolated from EACs have a contractile, myofibroblastic phenotype and promote EAC cell invasion *in vitro* (Transwell assays, *p* ≤ 0.05; organotypic culture, *p <* 0.001) and *in vivo* (*p* ≤ 0.05). *In vitro*, this pro-invasive effect is modulated through the matricellular protein periostin. Periostin is secreted by CAFs and acts as a ligand for EAC cell integrins αvβ3 and αvβ5, promoting activation of the PI3kinase–Akt pathway. In patient samples, periostin expression at the tumour cell–stromal interface correlates with poor overall and disease-free survival. Our study highlights the importance of the tumour stroma in EAC progression. Paracrine interaction between CAF-secreted periostin and EAC-expressed integrins results in PI3 kinase–Akt activation and increased tumour cell invasion. Most EACs contain a myofibroblastic CAF-rich stroma; this may explain the aggressive, highly infiltrative nature of the disease, and suggests that stromal targeting may produce therapeutic benefit in EAC patients.

## Introduction

Oesophageal cancer is associated with extremely poor overall survival; 60–70% of patients present with late-stage disease, too advanced for treatment with curative intent, and in those patients who are suitable for multimodal therapy, 5 year survival is only 35% [1]. This is partly due to the mechanically compliant oesophageal anatomy, which allows symptomless tumour expansion [2]. Additionally, tumour invasion and metastasis are facilitated by the absence of an outer serosal layer and the presence of a rich lymphatic plexus. Depth of invasion and nodal metastasis are both prognostic, and improving survival rates will require an increased understanding of the molecular mechanisms regulating local and regional tumour spread.

Although most cancer-related research has focused on tumour cells [3–5], accumulating evidence suggests that ‘normal’ cells within the tumour stroma (including fibroblasts, endothelial and immune cells) play a major role in tumour development and progression [6]. Stromal features are prognostic in many tumour types [7–11], and expression of stromal genes has been shown to be associated with poor outcome in oesophageal cancer [12].

Fibroblasts are probably the most abundant stromal cells in most cancers, and are recognised to play a role in the development and progression of a range of epithelial tumours [13]. Although fibroblasts within cancers comprise a phenotypically heterogeneous population, and the terminology is confusing [peritumour fibroblasts, cancer-associated fibroblasts (CAFs), ‘activated’ fibroblasts], ‘activated’ CAFs are commonly described as having a myofibroblastic phenotype; ie a secretory and contractile cell which expresses α-smooth muscle actin (α-SMA) [14,15]. These cells are responsible for the stromal desmoplasia observed in a number of solid cancers [16–18] and are associated with poor prognosis in several carcinoma types, including colorectal [10], breast [19], ovarian [20] and head and neck cancers [8]. CAFs regulate a number of tumour-promoting functions, including invasion [21] and angiogenesis [22], and may also affect tumour cell function by remodelling and generating tissue tension [23]. CAFs are predominantly derived from local fibroblasts, but it is clear that other cell types, including stellate cells, pericytes and circulating mesenchymal stem cells, can undergo myofibroblast transdifferentiation [24]. Irrespective of the progenitor cell, TGFβ1 signalling, in conjunction with increased mechanical resistance of the extracellular matrix (ECM), is central to the transdifferentiation process [25,26]. Notably, the stromal signature identified by Saadi and colleagues [12] in oesophageal cancer patients contained a predominance of inflammation and TGFβ-related genes.

To date, little is known regarding the role of the tumour microenvironment, and in particular CAFs, in the development and progression of EAC [27,28]. In this study we investigated the prognostic and functional role of CAFs in EAC and provide new insights into the role of periostin in these processes.

## Materials and methods

### Tissue and cell collection, maintenance and RNAi

Tissue was collected and stored with ethical agreement and informed consent; 09/H0504/66. Fibroblasts were extracted from normal oesophagus and oesophageal adenocarcinoma and subcultured as previously described [29]. siRNA-mediated silencing of periostin was carried out using INTERFERin transfection reagent and two commercially available siRNA sequences (for full details, see supplementary material, Supplementary materials and methods).

### Recombinant periostin

Periostin isoform 2 was subcloned and subsequently purified from Hek293F cells (for full details, see supplementary material, Supplementary materials and methods).

### Immunohistochemistry

Optimisation and staining of cohorts on full-face sections, using mouse monoclonal α-SMA (M085129-2, Dako, USA) and polyclonal rabbit anti-periostin (ab14041, Abcam, UK), was carried out on a Dako Link automated staining machine, according to the manufacturer's instructions. Sections were scored by a surgeon (TJU) and a pathologist (GT) blinded to patient outcome, using the following protocol: negative, no staining/<5%; moderate, patchy staining/5–50%; high, > 50%, as previously described [8].

### Western blotting

Cells (FLO-1 or primary fibroblasts) were treated under different conditions including ± fibroblast conditioned medium, ± recombinant periostin, ± TGFβ1, ± TGFβR1 kinase inhibitor IV (Calbiochem), ± LY294002 (Sigma) and ± αvβ3 and αvβ5 integrin antibodies (R&D Systems). Cells were treated with either TGFβ1 or integrin receptor inhibitors for 1 h before treatment with recombinant proteins (TGFβ1 and periostin) or clarified conditioned medium (for full details of antibodies used, experimental conditions and western blotting, see supplementary material, Supplementary materials and methods).

### Immunofluorescence

Immunofluorescence analyses were carried out as previously described [31]: primary antibodies, rabbit polyclonal anti-periostin (ab14041, Abcam) and mouse monoclonal anti-α-SMA (M085129.2, Dako); secondary antibodies, AlexaFluor 568 donkey anti-mouse IgG and AlexaFluor 488 goat anti-rabbit IgG (Molecular Probes).

### Organotypic cultures

Organotypic cultures were carried out as previously described [29,32]. NOFs or CAFs were co-cultured with either OE33 or FLO-1 cells for 14 days. Fibroblasts were pretreated with POSTN siRNA sequence 1 (Ambion) before embedding in organotypic cultures, and matured for 10 days. Organotypics treated with 10 µm LY294002 were matured for 14 days. Invasion was quantified and compared as previously described [33].

### Transwell invasion

Transwell invasion assays were carried out as previously described [32]: 24 h-conditioned medium from NOFs or CAFs (plated in identical numbers) ± POSTN siRNA pre-treatment, ± TGFβ1 pre-treatment. Addition of 5 µg/ml recombinant POSTN was used as the chemoattractant in the bottom chamber where indicated. Blocking antibodies for αvβ3 and αvβ5 integrins (10 µg/ml; R&D Systems) and PI3K inhibitor LY294002 (10 µm; Sigma) were added to the top chamber in combination with FLO-1 or OE33 cells for the duration of the experiment (72 h). All experiments were repeated three times, with four replicates/experiment. Normalisation was performed to the mean value of replicate 1 for all experimental conditions and data expressed as percentage invasion compared to the appropriate control (ie negative control siRNA- or vehicle-treated cells).

### Gel contraction

Gel contraction assays were carried out as previously described [8]: 0.5 × 10^6^ normal or CAFs (±POSTN siRNA knockdown) were seeded in collagen-1 gels.

### Tumour xenografts

OE33 or OANC1 cells (5 × 10^6^) ± NOFs or CAFs (1 × 10^6^) were injected subcutaneously into the flanks of SCID mice (one injection/mouse) in a 1:1 mixture of PBS and Matrigel™ (BD Biosciences; for full details of this model, see supplementary material, Supplementary materials and methods).

### Data acquisition and processing for network analysis

Weighted gene correlation network analysis (WGCNA) [34,35] was applied to microarray datasets generated on Affymetrix HG-U133A GeneChips from two independent studies (for full details of this analysis, see supplementary material, Supplementary materials and methods).

### Statistical analysis

Statistical analysis was performed with SPSS® v 19 (SPSS, Chicago, IL, USA). Survival of patients was plotted using the Kaplan–Meier method and analysed using the log-rank test, with patients censored at last follow-up. Multivariate Cox logistic regression was used to assess the relationship between α-SMA and standard pathological variables after resection. Kruskal–Wallis and Mann–Whitney U- and *t*-tests were used to compare groups, as appropriate; *p <* 0.05 was considered statistically significant for all tests (*p < 0.05, **p < 0.01).

## Results

### High α-SMA expression is common in EAC and correlates with poor overall survival following oesophageal resection

**Figure 1:**
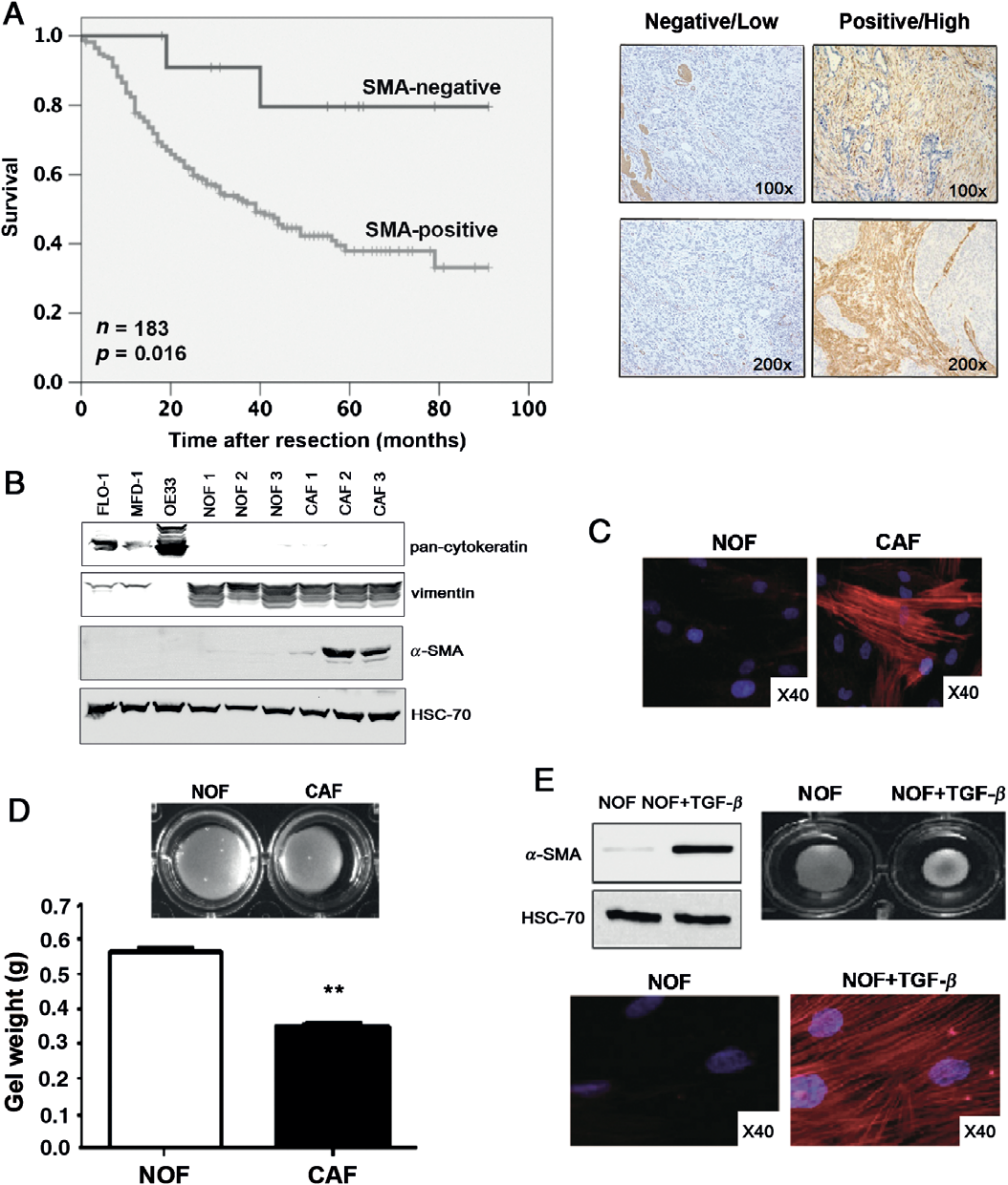
CAFs in the microenvironment are important in EAC. (A) Kaplan–Meier curve of overall survival after resection in patients with α-SMA-positive versus α-SMA-negative tumours; representative IHC images of negative/low and positive/high are in the right-hand panel. (B) Western blot for pan-cytokeratin and vimentin in FLO-1, OE33 and primary oesophageal NOFs and CAFs. (C) Immunocytochemistry for α-SMA in primary oesophageal NOFs and CAFs. (D) Photograph of a representative gel contraction assay at 72 h and gel weight with the incorporation of CAFs compared to NOFs (*p <* 0.01; *n =* 3). (E) Western blot and immunocytochemistry for α-SMA in NOFs and NOFs + TGFβ1, and a representative gel contraction assay

We used immunochemistry to examine the expression of α-SMA in the stroma of tumours from 183 patients following oesophageal resection (median follow-up = 4.5 years; 81 cancer-related deaths). Moderate/high expression of α-SMA (α-SMA-positive) was found in the majority of cases (*n =* 171). In 12 cases an α-SMA-negative stroma was observed, and this group of patients had a significant overall survival advantage [Figure 1A; mean survival α-SMA negative = 78.66 months (median not yet reached) versus α-SMA-positive = 48.14 months; *p =* 0.016 (median 39 months; 95% CI 29.2–48.8)]. The α-SMA-negative group was further analysed to exclude the possibility that it was biased towards early-stage disease; this was not the case. The pathological stages according to the AJCC (7th edn) classification of EAC [36] for the patients in this group were: stage 0, one patient; stage IA, three patients; stage IIA, one patient; stage IIB, two patients; stage IIIA, two patients; stage IIIB, one patient; and stage IIIC, two patients. When α-SMA expression was compared against other commonly used indicators of prognosis following surgery (TNM stage [36,37] and resection clearance: R status) in a Cox regression model, α-SMA expression was the most predictive factor analysed: hazard ratio (HR) 7.1 (1.7–29.4); *p =* 0.007 (Table1). Full details of the patient demographics are shown in Table S1 (see supplementary material).

**Table 1:** Cox regression analysis of α-SMA expression compared against other commonly used indicators of prognosis following oesophageal resection for EAC

Pathologicalvariable	Hazardratio	95% Confidence interval		Significance(*p*)
		Lower	Upper	
	1.49	1.14	1.95	0.003
pT stage				
pN stage	1.78	1.45	2.18	< 0.001
pM stage	1.57	0.62	3.96	0.343
Positive resection margin (R1)	1.31	0.81	2.11	0.271
α-SMA-positive stroma	7.07	1.70	29.39	0.007

### *Ex vivo* analysis of primary oesophageal CAFs reveals a myofibroblast phenotype that can be induced by TGFβ

CAFs were cultured directly from tumour tissue and NOFs from macroscopically and microscopically normal areas of mucosa at the proximal resection margin. NOFs and CAFs were characterised using cell-type specific markers, confirming a fibroblastic phenotype [vimentin-positive, cytokeratin-negative, CD31-negative (Figure 1B; see also supplementary material, Figure S1A) and desmin-negative (data not shown)]. CAFs expressed high levels of α-SMA, confirming a myofibroblast phenotype (Figure 1B). In an additional panel of primary fibroblasts, seven of eight CAFs expressed high levels of α-SMA compared with low or absent α-SMA levels in NOFs, in keeping with the immunohistochemical analysis of paraffin-embedded tissues (see supplementary material, Figure S1B). α-SMA stress fibre formation was observed in CAFs but not NOFs (Figure 1C). In gel contraction assays there was a significant reduction in gel size and weight in the presence of CAFs compared to NOFs (weight, *p <* 0.01), demonstrating the ability of CAFs to contract an *in vitro* ECM (Figure 1D).

Gene ontology analysis of the stromal signature in the progression of pre-invasive to invasive disease in EAC has previously identified TGFβ signalling as a critical pathway in this transition [12]. Treatment of NOFs with TGFβ1 for 72 h resulted in high levels of α-SMA expression, stress fibre formation and increased gel contraction (gel weight NOFs 0.21 g versus NOFs + TGFβ1 0.15 g; *p <* 0.01), confirming myofibroblast transdifferentiation (Figure 1E). In addition, exposure of NOFs to conditioned medium from FLO-1 cells for 72 h resulted in a TGFβ1-dependent up-regulation of α-SMA (see supplementary material, Figure S1C).

### CAFs promote EAC cell invasion *in vitro*

**Figure 2:**
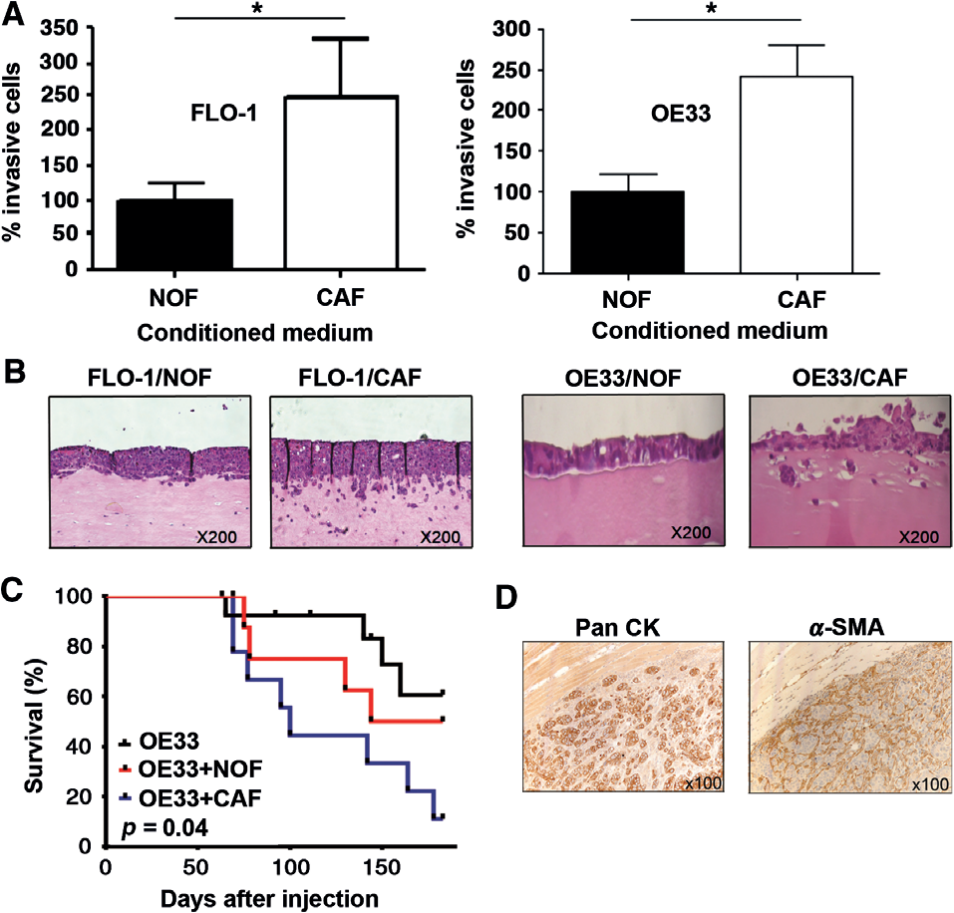
CAFs promote EAC cell invasion *in vitro* and tumour growth *in vivo*. (A) Transwell invasion assays comparing the effect of NOFs and CAFs conditioned medium on invasion of the EAC cell lines FLO-1 and OE33. (B) Organotypic models of EAC comparing the effect of NOFs and CAFs to promote invasion of FLO-1 and OE33. (C) Kaplan–Meier curves of overall survival of mice injected with OE33 alone or in combination with NOFs or CAFs. Mice were culled when the tumour volume reached 500 mm^3^. (D) Immunohistochemistry for Pan-CK and α-SMA in tumour xenografts containing OE33 and CAFs

To determine whether CAFs secrete factors that promote EAC cell invasion, Transwell invasion assays were performed using EAC cell lines FLO-1 and OE33. Conditioned medium from NOFs or CAFs was used as a chemoattractant in the lower chamber of the Transwell. CAFs-conditioned medium promoted a > two-fold increase in invasion of both EAC cell lines (*p <* 0.05; Figure 2A). The proliferation of FLO-1 cells exposed to either NOF- or CAF-conditioned medium was assessed by MTS assay, and no difference was observed (see supplementary material, Figure S2B). Using organotypic cultures as a more physiologically relevant model of tumour cell invasion [29], we further explored the role of EAC CAFs in this process. Invasion in these models was quantified and compared, taking into account the average depth of tumour invasion and the number and area of invading tumour islands [33]. Minimal invasion was seen in NOFs-containing gels, but in the presence of CAFs, EAC cell invasion was significantly increased for both tumour cell lines [mean total area of invasion, arbitrary units (*n =* 3), FLO-1 + NOFs 2670.3 versus FLO-1 + CAFs 3862.3; *p <* 0.001: OE33 + NOFs 501 versus OE33 + CAFs 6238.3; *p <* 0.001: Figure 2B; see also supplementary material, Figure S2A].

### CAFs promote tumour growth *in vivo*

The ability of CAFs to support tumour growth *in vivo* was assessed using a xenograft model. The cell line FLO-1 was non-tumourigenic in this model and therefore experiments were performed using OE33 cells. Xenografts from OE33 cells injected subcutaneously, either alone (*n =* 15) or mixed with NOFs (*n =* 9) or CAFs (*n =* 9), did not grow uniformly and therefore the mice were culled when the tumour volume had reached 500 mm^3^, and groups were compared by Kaplan–Meier analysis. Mice receiving co-injections of OE33 cells and CAFs were culled significantly earlier (median survival OE33 + CAFs = 100 days versus OE33 + NOFs = 163.5 days; *p =* 0.04; Figure 2C). Immunostaining for α-SMA confirmed the retained presence of CAFs in these tumours surrounding cytokeratin-positive tumour cells (Figure 2D). Mice injected subcutaneously with an additional EAC cell line, OANC1 + CAFs (*n =* 4), developed tumours that grew more rapidly than those injected with OANC1 + NOFs (*n =* 4) (see supplementary material, Figure S2C).

### Periostin is secreted by CAFs and promotes tumour cell invasion

Saadi and colleagues [12] recently described a stromal gene expression signature that is predictive of outcome in EAC. To complement this differential expression study, we applied WGCNA [38] to a publicly available EAC microarray dataset [39]. Seven modules of highly co-expressed genes were identified by unsupervised hierarchical clustering on the basis of topological overlap (TO) and labelled by colour (Table2). The co-expression network was strongly preserved in an independent dataset [40].

**Table 2:** Correlation matrix of seven modules (labelled by colour) of highly co-expressed genes that have been identified by unsupervised hierarchical clustering on the basis of topological overlap

Module colour	Number of genes	Module preservation (Zsummary)	Module Gene Ontology	Bonferoni (*p*)
Black	788	10	Intracellular transport	5.20E-11
Blue	1143	50	Skin development	1.10E-17
Brown	384	16	Cell cycle process	7.20E-06
Cyan	271	29	Nucleotide metabolic process	6.50E-06
Grey	745	13	DNA metabolic process	4.50E-15
Light green	467	39	Antigen processing and presentation	1.20E-06
Pink	202	23	Extracellular matrix organization	2.70E-34

The Zsummary is a composite statistic of density and connectivity preservation for the module: < 2, no preservation; 2–10, weak to moderate preservation; > 10, strong evidence of preservation.

**Figure 3:**
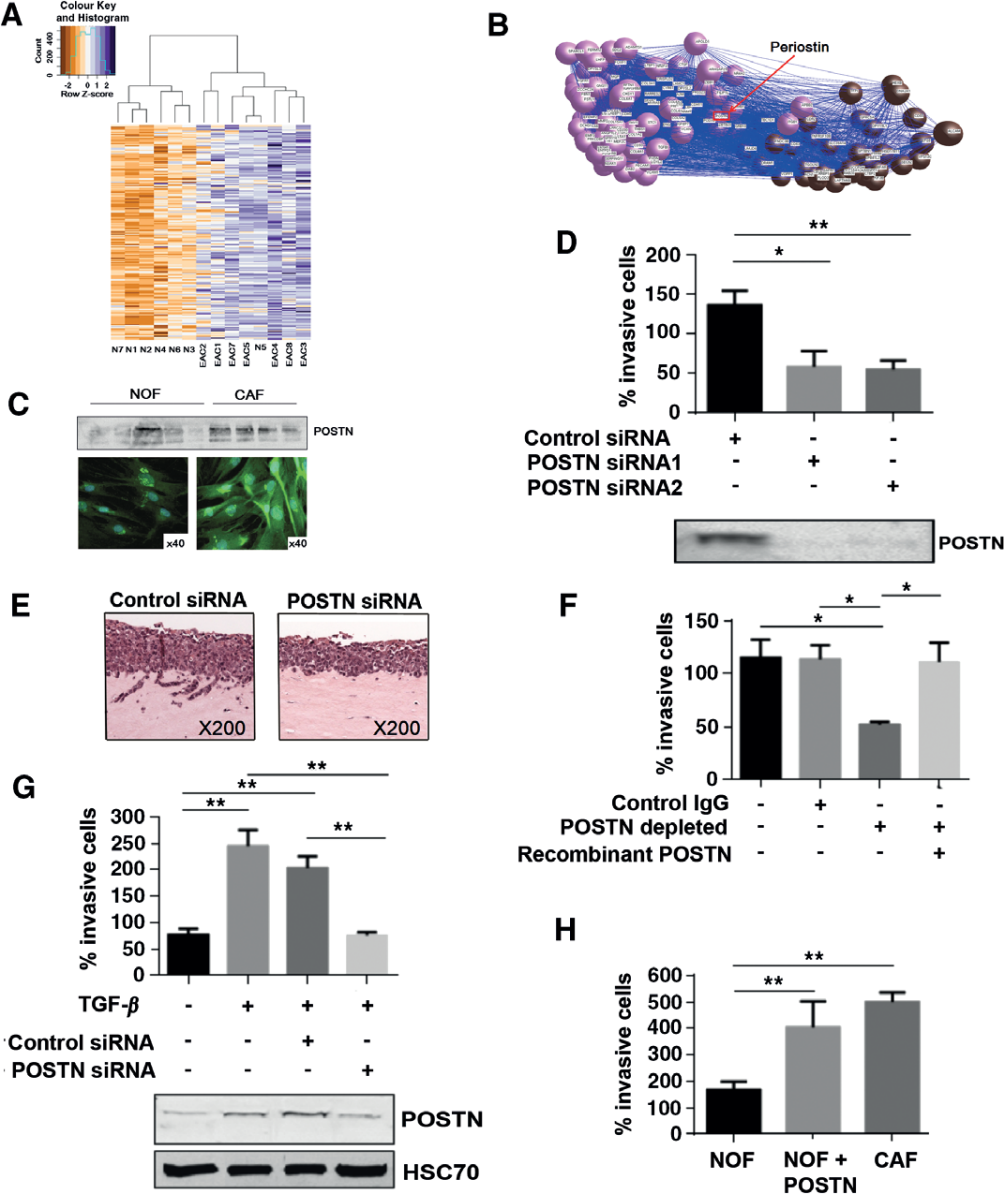
Periostin is secreted by CAFs and promotes EAC cell invasion. (A) Unsupervised hierarchical clustering of the ECM module genes differentiated between normal oesophageal epithelium and EAC. (B) Exported network weights in BioLayout Express^3D^, graphically representing periostin as a central gene within the ECM module, with many connections to other ECM module genes and genes associated with the cell-cycle process. (C) Western blot of conditioned medium and immunocytochemistry, comparing the expression and secretion of periostin in NOFs and CAFs. (D) Transwell invasion assays, demonstrating the invasion of FLO-1 in response to CAF-conditioned medium ± periostin silencing, as indicated (*p < 0.05; **p < 0.001), with western blot of conditioned medium normalized to cell number confirming periostin knockdown. (E) Organotypic cultures of FLO-1 and CAFs, with and without periostin silencing. (F) Transwell invasion assays of FLO-1 towards CAF-conditioned medium, with or without periostin immunodepletion and rescue with recombinant periostin, as indicated (*p < 0.05). (G) Transwell invasion assay of FLO-1 towards conditioned medium from NOFs or NOFs + TGFβ1, with and without periostin silencing, and western blot analysis of periostin expression under the same conditions (**p < 0.001). (H) Transwell invasion assay of FLO-1 towards conditioned medium from NOFs ± recombinant periostin or CAFs, as indicated (**p < 0.001)

ToppGene analysis indicated that the pink module was highly enriched for genes associated with fibroblasts (GSM777043; Bonferroni adjusted *p =* 3.1 × 10^−83^). Concomitant with this finding, the top Gene Ontology (GO) category for the pink module was ‘extracellular matrix organisation’ (Table2); among the enriched genes were α-SMA, fibrillin 1 and periostin. Unsupervised hierarchical clustering of the ECM module genes differentiated between normal oesophageal epithelium and EAC (Figure 3A). These observations are consistent with the ECM module representing genes expressed by a CAFs-rich stroma.

The relative importance of each gene within its respective module can be estimated by a module membership measure (KME) [38]. Periostin had a high KME value (0.87), denoting centrality and importance within the ECM module; a full list of KME values is provided in Table S2 (see supplementary material). Of the top 86 nearest neighbours of periostin (TO threshold ≥ 0.15; Figure 3B), 52 of the genes were annotated for the GO categories ECM organization (adj*P =* 4.1 × 10^−27^), biological adhesion (adj*P =* 7.0 × 10^−17^) and locomotion (adj*P =* 3.2 × 10^−8^).

We therefore examined whether the tumour-promoting effects of CAFs are modulated through periostin. Conditioned medium from CAFs contained higher levels of periostin compared to NOFs by western blot and immunocytochemistry (Figure 3C). siRNA-silencing of POSTN in CAFs suppressed periostin expression and resulted in a > 50% suppression of tumour cell invasion in Transwell assays (*p <* 0.05; Figure 3D). POSTN silencing in CAFs also resulted in complete loss of invasion by EAC cells in organotypic culture (mean total area of invasion, arbitrary units; *n =* 3; control siRNA 1187.7 versus POSTN siRNA 3581; *p <* 0.001; Figure 3E). To further confirm that the inhibition of tumour cell invasion was directly modulated through reduced periostin expression, we immunodepleted periostin from CAFs-conditioned medium and observed a similar reduction in invasion (*p <* 0.05). This was restored by addition of recombinant periostin (Figure 3 F). Fibroblast proliferation after periostin silencing was not significantly different to control siRNA-treated cells (data not shown).

We had previously found that TGFβ1 treatment of NOFs generated a myofibroblastic phenotype (Figure 1D, E). TGFβ1-treated NOFs also up-regulated POSTN (Figure 3G) and promoted EAC invasion (*p <* 0.01). Periostin silencing did not affect the up-regulation of α-SMA in TGFβ1-treated NOFs (see supplementary material, Figure S3F). The increase in EAC invasion was again periostin-dependent and abrogated by POSTN down-regulation. Furthermore, the addition of recombinant periostin to NOF-conditioned medium significantly increased EAC cell invasion in Transwell assays to levels comparable to CAF-conditioned medium (Figure 3H).

### Periostin treatment of EAC cells results in integrin- and PI3K-dependent activation of Akt

**Figure 4:**
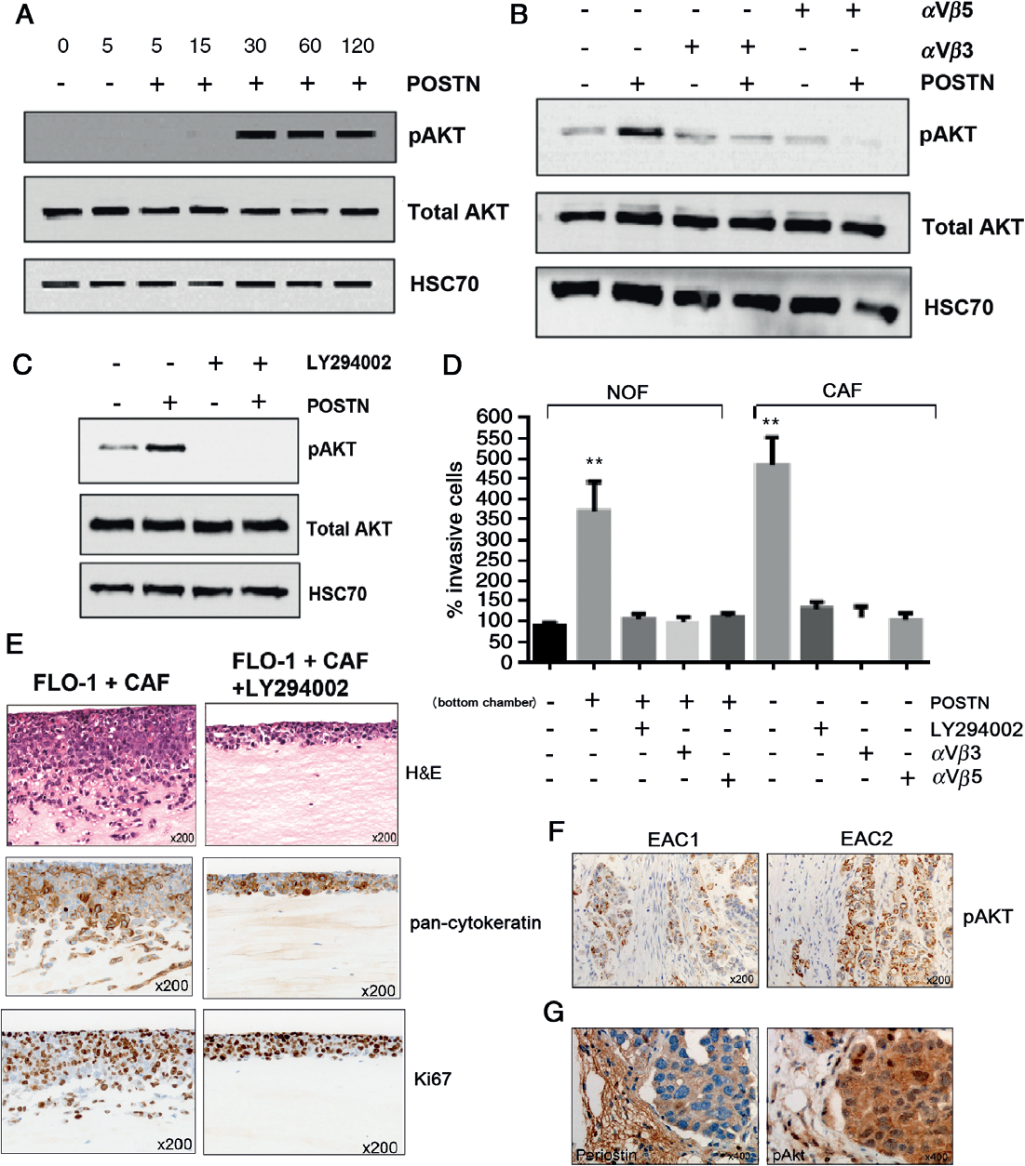
Periostin-dependent invasion of EAC cells is suppressed through inhibition of αvβ3 and αvβ5 integrins and PI3K. (A) Western blot for total and phospho-Akt in FLO-1 up to 120 min after stimulation with recombinant periostin. (B) Western blot for total and phospho-Akt in FLO-1, 30 min after periostin stimulation, in the presence or absence of blocking antibodies against integrins αvβ3 or αvβ5, as indicated. (C) Western blot for total and phospho-Akt in FLO-1, 30 min after periostin stimulation, in the presence or absence of the PI3K inhibitor LY294002. (D) Transwell invasion assays of FLO-1 towards NOFs or CAFs conditioned medium under varying conditions. Invasion towards NOF-conditioned medium is used as the internal control. Periostin was added to the NOF-conditioned medium and either LY294002 or blocking antibodies against integrins αvβ3 or αvβ5 were added to the FLO-1 cells, as indicated. (E) Organotypic models of FLO-1 + CAFs in the presence or absence of LY294002, including staining by H&E, pan-cytokeratin and Ki67. (F) Immunohistochemistry for p-Akt in representative human EAC tumours with strongest staining adjacent to CAFs. (G) Immunohistochemistry for periostin and p-Akt in tumour xenografts containing OE33 and CAFs

We examined potential periostin-dependent downstream signalling pathways in EAC cells and found Akt to be consistently phosphorylated following periostin treatment. PI3K–Akt signalling has been reported to be a critical regulator of cell motility in several cancer types, including EAC, and we explored this response in more detail. Recombinant periostin was added to tumour cells and phosphorylation of Akt was observed at 30 min post-stimulation, which persisted for at least 120 min (Figure 4A). Periostin is a ligand for several integrins, including αvβ3 and αvβ5, and so we examined whether periostin-dependent Akt activation is modulated through integrin binding. After confirming the presence of αvβ3 and αvβ5 on the surface of EAC cells by western blot and flow cytometry (see supplementary material, Figure S3A, B), we found that phosphorylation of Akt in response to periostin was suppressed by blocking antibodies directed against αvβ3 or αvβ5 (Figure 4B). The suppression of Akt phosphorylation in periostin-treated cells, in the presence of the PI3K inhibitor LY294002 confirmed that Akt phosphorylation was PI3K-dependent (Figure 4C). Similar effects on Akt activation were observed in FLO-1 cells after 30 min treatment with CAFs-conditioned medium (see supplementary material, Figure S3E).

### Periostin-dependent invasion is suppressed through inhibition of αvβ3, αvβ5 and PI3K

We repeated Transwell invasion assays to examine the effect of inhibiting αvβ3/αvβ5–PI3K–Akt signalling in EAC cells. NOF-conditioned medium supplemented with recombinant POSTN or CAF-conditioned medium promoted EAC invasion (>3.5-fold; Figure 4D) and this was inhibited by the addition of αvβ3- or αvβ5-blocking antibodies, or a PI3K inhibitor, LY294002, to the tumour cells. Invasion of EAC cells in organotypic culture containing CAFs was significantly reduced in the presence of LY294002 (mean total area of invasion, arbitrary units (*n =* 3), FLO-1 + CAFs 11961 versus FLO-1 + CAFs + LY294002 338.7; *p <* 0.001; Figure 4E). Proliferation of FLO-1 cells was not affected by treatment with LY294002 in organotypic assays, shown by Ki67 staining (Figure 4E), percentage of Ki67-positive cells in 10 high-power fields (vehicle = 87 ± 5, LY294002 = 89 ± 3). This was confirmed by MTS assays across a range of concentrations (see supplementary material, Figure S3C). An increase in caspase-8 expression (not cleavage) was observed (see supplementary material, Figure S3D). To confirm Akt activation in human EAC tumours, we performed immunohistochemistry (IHC) and observed pAkt in tumour cells, with the strongest staining adjacent to CAFs (Figure 4 F). High stromal periostin expression was observed in the mouse xenograft model containing OE33 and CAFs, and activation of Akt was confirmed in this model by high pAkt staining in adjacent tumour cells (Figure 4G).

### Periostin expression predicts survival after resection for EAC

**Figure 5:**
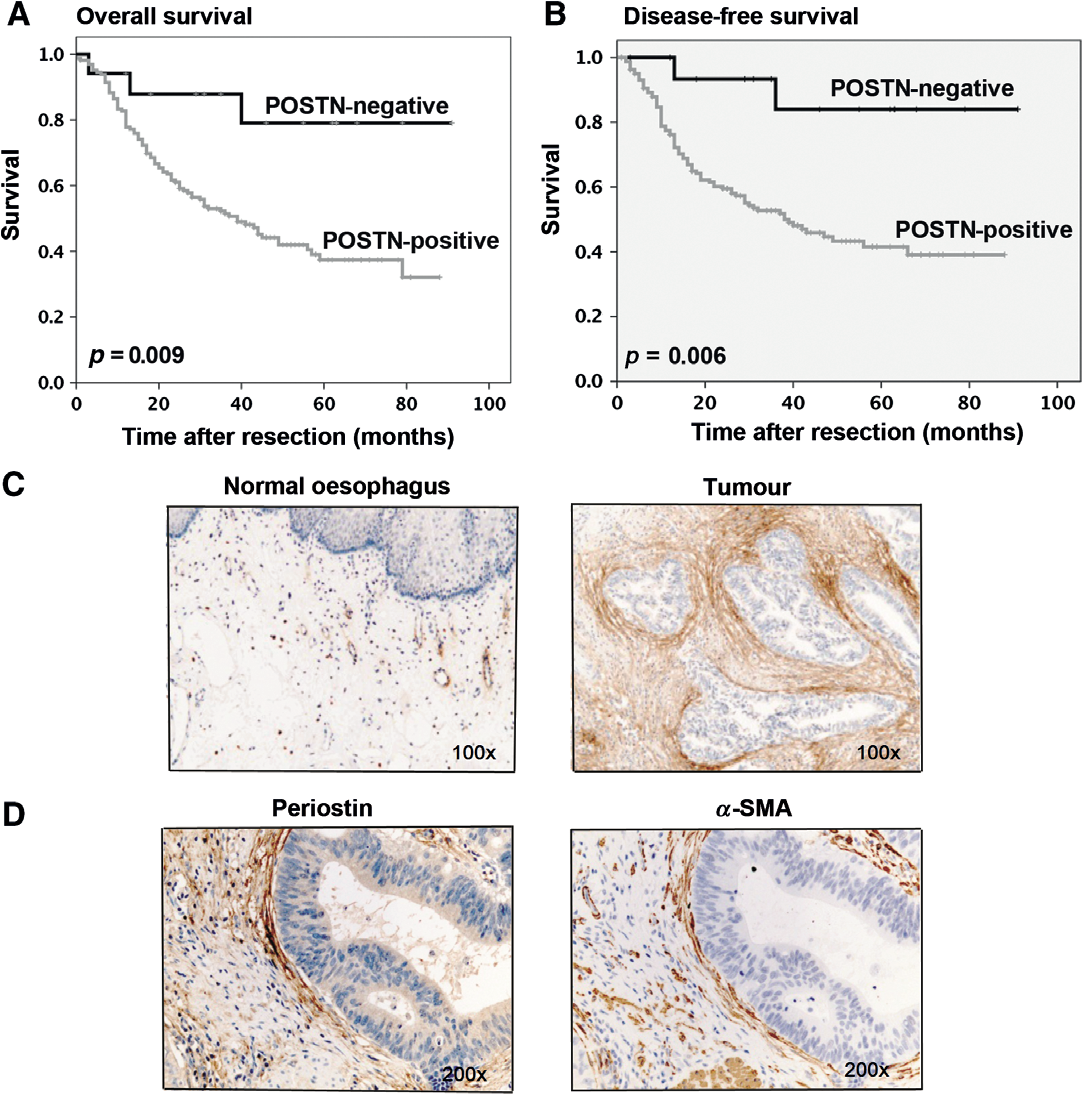
Stromal periostin expression correlates with poor survival in EAC. (A) Kaplan–Meier curves of overall survival after resection in patients with periostin-positive versus periostin-negative tumours. (B) Kaplan–Meier curves of disease-free survival after resection in patients with periostin-positive versus periostin-negative tumours. (C) Immunohistochemistry for periostin expression in human normal oesophagus (limited to the walls of blood vessels) and EAC. (D) Immunohistochemistry to compare expression patterns of periostin and α-SMA in EAC

Having established the importance of periostin to CAFs-induced EAC cell invasion *in vitro*, we examined our EAC patient cohort to confirm the presence of stromal periostin by IHC. Similar to α-SMA expression, moderate/high expression of periostin was observed in the majority of cases (*n =* 166; periostin-positive), with periostin localised to the cancer cell–stromal interface (Figure 5C, D), corresponding to areas of high α-SMA expression (Figure 5D) in keeping with a myofibroblastic CAFs origin. Periostin expression was limited to blood vessel walls in normal oesophageal mucosa (Figure 5C).

In 17 cases there was no/minimal periostin staining (periostin-negative) and this group of patients had a significant overall survival advantage when compared with periostin-positive patients [Figure 5A; mean survival periostin-negative = 76.45 months (median not yet reached) versus periostin moderate/high = 46.80 months (median 39 months); *p =* 0.009]. We also examined the effect of periostin expression on disease-free survival (Figure 5B). Patients with no periostin staining in their resected tumours had a mean time to recurrence of 80.67 months (median not yet reached). Patients with high levels of periostin staining in the resected tumours experienced the shortest mean time to recurrence of 47.45 months (median 38 months; *p =* 0.006).

## Discussion

The traditional model of EAC development describes progressive cellular dysplasia and accumulation of mutations in response to persistent acid reflux and inflammation [41]. Whole-genome and exome-sequencing studies are beginning to define the complexity of EAC, but suggest that the mutational burden may already be established in non-dysplastic BE [42–44], meaning that the drivers for disease progression lie beyond gene mutations in the epithelial cells and that the stromal tissues play a critical role in disease development and progression.

However, very little is known about the role of the tumour microenvironment/stroma in oesophageal cancer [27,28]. CAFs are a heterogeneous cell type, and no single marker reliably identifies all CAFs. They are most commonly identified by α-SMA expression, indicative of an 'activated' myofibroblast-like phenotype. We found that the vast majority of patients with EAC have tumours with high or moderate levels of stromal α-SMA; this predicts poor survival and may account, in part, for the aggressive nature of the disease. Retrospective studies have intrinsic limitations and the uneven distribution of SMA expression in our cohort raises the possibility of a type 1 error; however, this may reflect the importance of the CAFs phenotype in EAC, which is clearly supported by the *in vitro* and *in vivo* data. EAC CAFs cultured from primary tumours have an α-SMA-positive, contractile, myofibroblastic phenotype; they express a range of mesenchymal and fibroblast-specific markers, and promote EAC invasion *in vitro* and tumour growth *in vivo*. We provide evidence of a potential mechanism by showing that the invasion-promoting effect of CAFs is modulated, in part, through periostin, a secreted matricellular protein that plays an important role in tissue remodelling and collagen fibrillogenesis, interacting with ECM proteins such as fibronectin, tenascin C and collagen V, and with cell surface receptors, most notably integrins, including αvβ3, αvβ5 and α6β4 [45–47]. We found that CAFs express high periostin levels *in vitro* and *in vivo*, and activate tumour cell PI3K–Akt signalling through integrin binding. Similar to α-SMA, periostin expression also predicts poor outcome in EAC patients.

A stromal gene-expression signature containing periostin has previously been shown to predict outcome in EAC [12]. However, most cancer studies have found periostin to be of tumour cell origin, with over-expression documented in a range of tumours, including lung, breast, ovary and pancreas [48]. Its functional role in cancer is not clear, although expression correlates with poor outcome in several cancers, suggesting a tumour-promoting effect, and, consistent with this, periostin has been reported to modulate a number of the hallmarks of malignancy [48]. Metastasising breast cancer cells induce periostin secretion in the cancer stem cell niche and require continued stromal periostin expression for cancer stem cell maintenance [49]. Importantly, disruption of the interaction between periostin and the integrin receptors αvβ3 and αvβ5 by periostin-binding DNA aptamers blocked signal transduction and reduced primary tumour growth and metastasis in an orthotopic breast cancer mouse model [50], suggesting that targeting periostin has therapeutic potential. Periostin is involved in remodelling of the ECM to support tumour development, invasion and metastasis [48]. Periostin has also been identified as a key component of an invasive signature in a three-dimensional (3D) organotypic model of oesophageal squamous cell carcinoma [51]. We have confirmed the importance of periostin in EAC biology. Applying WGCNA to publicly available EAC microarray datasets has identified periostin to have a 'nodal' position within ECM gene expression in EAC, with close associations with genes involved in cancer cell invasion, adhesion and locomotion.

Periostin contains a FAS1 domain that allows binding of αv-integrins and glycosaminoglycans *in vivo*. Interaction with these cellular receptors can lead to diverse downstream signalling effects in a context-dependent manner (NF-*κ*B, STAT3, PI3K–Akt and FAK), controlling the expression of many genes (including α-SMA, collagen and fibronectin) [52]. We found that periostin treatment of EAC cells results in integrin- and PI3K-dependent activation of Akt, and furthermore that periostin-dependent EAC cell invasion is suppressed through inhibition of αvβ3, αvβ5 and PI3K. These findings suggest not only that periostin is very likely to be important for EAC cell invasion *in vivo*, but also that this is mediated by integrin–PI3K–Akt signalling. The PI3K signalling pathway integrates intra- and extracellular signals to modulate a multitude of downstream responses, effected by multiple substrates [53]. PI3K activation in response to integrin signalling is well documented in cancer, and there are some reports of Akt activation leading to tumour cell invasion in response to integrin-mediated periostin signalling in epithelial tumours, eg integrin α5β1 in cholangiocarcinoma [47]. Canonical PI3K–Akt signalling is frequently dysregulated in cancer and can determine cell growth, proliferation, angiogenesis, migration and invasion [54]. The fact that periostin has been implicated in many of the same processes suggests that the PI3K–Akt pathway may act as a node for periostin signalling in cancer. Our findings suggest that in EAC it may be possible to target periostin–intergrin–PI3K–Akt signalling at multiple levels with new and existing therapeutics. Further work is needed to confirm this hypothesis. For instance, it will be important to know whether periostin is prognostic in pretreatment biopsies from EAC, and whether or not the presence of periostin predicts response to neoadjuvant chemotherapy.

In conclusion, our study highlights the importance of the tumour stroma in EAC progression. The majority of EACs contain a prominent myofibroblastic (α-SMA-positive) CAFs-rich microenvironment, and stromal α-SMA expression is more significantly prognostic than conventional histopathological criteria. CAFs isolated from EAC have a functional myofibroblastic phenotype, and promote tumour cell invasion *in vitro* and growth *in vivo*, signalling to EAC cells via secretion of the ECM protein, periostin. Disruption of periostin signalling via integrin receptors and the PI3K–Akt pathway leads to abrogation of cancer cell invasion. This opens the possibility of targeting CAFs and their paracrine signals as treatment strategies in oesophageal cancer.

## Abbreviations

AJCC: American Joint Committee on Cancer
BE: Barrett's oesophagus
CAF: cancer-associated fibroblast
EAC: oesophageal adenocarcinoma
ECM: extracellular matrix
IHC: immunohistochemistry
NOF: normal oesophageal fibroblast
POSTN: periostin
PI3K: phosphoinositide 3-kinase
α-SMA: smooth muscle actin
TGF: transforming growth factor
TNM: tumour node metastasis.

## Supporting Information on the Internet

The following supplementary material may be found in the online version of this article:

Supplementary materials and methods

Figure S1 Further characterization of primary oesophageal fibroblasts

Figure S2 Additional organotypic and mouse xenograft model

Figure S3 Integrin αvβ3 and αvβ5 expressions were analysed in FLO-1 and OE33 cells using western blot, FACs and MTS proliferation assay

Table S1 Clinicopathological characteristics of patient cohort for immunohistochemistry

Table S2 KME values for all of the proteins clustered in the ECM module
