# Supplementary material for: Cancer-associated fibroblasts predict poor outcome and promote periostin-dependent invasion in oesophageal adenocarcinoma
Source: J Pathol. 2015 Jan 8;235(3):466–77. doi: 10.1002/path.4467 (PMC4312957; doi:10.1002/path.4467)
Supplement: Supplementary file 1 [file path0235-0466-sd1.doc]

<**Supplementary material>**

+A: **Supplementary materials and methods**

+B: Cell culture

FLO-1 (EACC), OE33 (EACC), OANC1 and primary cells were maintained in Dulbecco’s modified Eagle's medium (DMEM; Invitrogen) or Roswell Park Memorial Institute medium (RPMI; Invitrogen) supplemented with 10% v/v fetal calf serum (FCS; Autogen Bioclear), 2 mm l-glutamine and 100 μg/ml penicillin–streptomycin (Invitrogen).

+B: SiRNA transfections

Commercially available sequences 1 (5′-GGCUUUGCACAUUUCUAUAtt-3′, 3′-ttCCGAAACGUGUAAAGAUAU-5′) and 2 (5′-GCUCUUAUGAAGUACCACAtt-3′, 3′-ttCGAGAAUACUUCAUGGUGU-5) (Ambion) were transfected into primary fibroblasts at 50% confluence (cells were seeded 24 h before transfection, 5 × 10^5^/well of a six-well plate), using INTERFERin (Polyplus) according to the manufacturer’s instructions. The cells were cultured for 72 h to achieve optimal knockdown; negative control 1 siRNA (Ambion) was used for experimental controls.

+B: Western blotting

The antibodies used were: mouse monoclonal anti-*α*-SMA (M085129-2, Dako), mouse monoclonal anti-HSC-70 (sc-7298, Santa Cruz, USA) and rabbit polyclonal anti-Periostin (ab14041, Abcam, UK), rabbit polyclonal anti-pAKT (4060, Cell Signaling, USA), rabbit polyclonal anti-total AKT (9272, Cell Signaling), human monoclonal anti-integrin *α*v*β*3 monoclonal (MAB3050, R&D Systems, USA), human monoclonal anti-integrin *α*v*β*5 monoclonal (MAB2528, R&D Systems), mouse monoclonal anti-CD31 (3528, Cell Signaling), polyclonal rabbit anti-Cytokeratin (z0622, Dako), mouse monoclonal anti-vimentin (m7020, Dako) and mouse anti-human caspase-8 (551244, BD Pharmingen). Adherent cells (FLO-1 or primary fibroblasts) were treated under different conditions, including ± fibroblast-conditioned medium, ± recombinant periostin, ± TGF*β*1, ± TGF*β*R1 kinase inhibitor IV (Calbiochem, USA), ± LY294002 (Sigma, USA) and ± *α*v*β*3 and *α*v*β*5 integrin antibodies (R&D Systems). Cells were pretreated with either TGF*β*1 or integrin receptor inhibitors for 1 h before treatment with recombinant proteins (TGF*β*1 and periostin) or clarified conditioned medium. Cells for pAKT expression were harvested by scraping at 4°C into phosphate-buffered saline (PBS) after an initial PBS wash and pelleted by centrifugation at 1000 rpm. All other cells were harvested by trypsin digestion after an initial PBS wash before pelleting by centrifugation. Cell lysis was carried out for 15 min at 4°C in 50 μl RIPA buffer (0.75 m NaCl, 5% NP40, 2.5% deoxycholic acid, 0.5% SDS, 0.25 m Tris, pH 8.0). Lysates were clarified by centrifugation at 8000 × *g* for 5 min. Fibroblasts or FLO-1 cells were cultured in serum-free DMEM for 24 h before the conditioned medium was harvested at 4°C, clarified by centrifugation and the supernatant concentrated, using Amicon Ultra-4-centrifugal 10 kDa filters (Millipore). Protein samples were quantified using the Bradford protein assay reagent. Protein (20 μg) or concentrated cell culture medium (20 μl; adjusted in SDS loading dye for cell number when conditioned medium was removed) were resolved using SDS–polyacrylamide gel (PAGE) electrophoresis and transferred to Hybond–ECL membranes (GE Healthcare, UK). Blocking and antibody incubations were done in 3% low-fat milk in PBS–0.025% Tween 20, and washes were in PBS–0.1% Tween 20. Detection of horseradish peroxidase-labelled secondary antibody was done with Supersignal (Pierce), and images were collected using a CCD camera (ChemiDoc-it® imaging system, UVP).

+B: Proliferation assays

MTS assays (Promega) were carried out according to the manufacturer’s instructions: 96-well plates were seeded with 1000 cells/well 24 h before different conditions were added [LY294002 (Sigma) or fibroblast-conditioned medium]. The cells were then incubated for 72 h before MTS reagent was added.

+B: Tumour xenografts

OE33 or OANC1 (5 × 10^6^) ± NOFs or CAFs (1 × 10^6^) were injected subcutaneously into the flanks of SCID mice (one injection/mouse) in a 1:1 mixture of PBS and Matrigel™ (BD Biosciences). Tumour growth was measured every 2–3 days, using electronic calipers, and tumour volumes were determined using the ovoid formula: (length × width^2^)/2. Tumour xenografts were analysed by Kaplan–Meier, where the end-point was defined as tumour volume of 500 mm^3^ and tumour growth data were plotted as growth curves, with data points representing the mean tumour volume ± SEM for each group. The project was approved by the Animal Experimentation Ethics Committee, Peter MacCallum Cancer Centre, Australia, and the experiments were performed in accordance with the *Australian Code of Practice for the Care and Use of Animals for Scientific Purposes*.

+B: Data acquisition and processing for network analysis

Weighted gene correlation network analysis (WGCNA) was applied to microarray datasets generated on Affymetrix HG-U133A GeneChips from two independent studies: (a) microarrays assessing gene expression from eight individuals with matched normal oesophageal epithelium, BE and EAC (GSE1420) ; and (b) 69 microarrays comprising oesophageal squamous epithelium from 19 healthy subjects, nine cases of ESCC, 20 specimens from patients with BE and 21 cases of EAC (GSE26886) . CEL files were imported into the R/Bioconductor package ‘affy’ and normalized using the RMA algorithm . Outlier subjects were identified and excluded, using a sample network connectivity statistic described by Oldham *et al*  and implemented in the R function SampleNetwork.

WGCNA package in R was performed on the GSE1420 dataset. Briefly, a correlation matrix was obtained by calculating the by-weight mid-correlations between all variable probe-sets across all samples. Next, the adjacency matrix was calculated by raising the absolute values of the correlation matrix to the power of 12. Topological overlap (TO) was then calculated for the 4000 most connected genes (among the 12 496 most varying annotated probe-sets). Finally, the genes were hierarchically clustered, using 1–TO as the distance measure, and modules were determined using a dynamic tree-cutting algorithm. The network was graphically depicted by exporting the TO weights into the program BioLayout Express^3D^ .

+B: Recombinant periostin

A full-length cDNA clone for human periostin isoform 2 was obtained from Source BioScience UK (IMAGE ID 40007999) and the DNA sequence corresponding to amino acids 22–779 was subcloned into the vector pFUSE–hIgG1–Fc2 (InvivoGen) with the addition of a C-terminus HA tag, transiently transfected into 293F cells using 293Fectin (Invitrogen) and the cells were cultured for 3–4 days. Protein was purified from the supernatant on an anti-HA agarose (Sigma) column and eluted using 1 mg/ml HA peptide prior to dialysis into PBS and storage at –70°C.
