# Supplementary figures and images for: Cancer-associated fibroblasts predict poor outcome and promote periostin-dependent invasion in oesophageal adenocarcinoma
Source: J Pathol. 2015 Jan 8;235(3):466–77. doi: 10.1002/path.4467 (PMC4312957; doi:10.1002/path.4467)

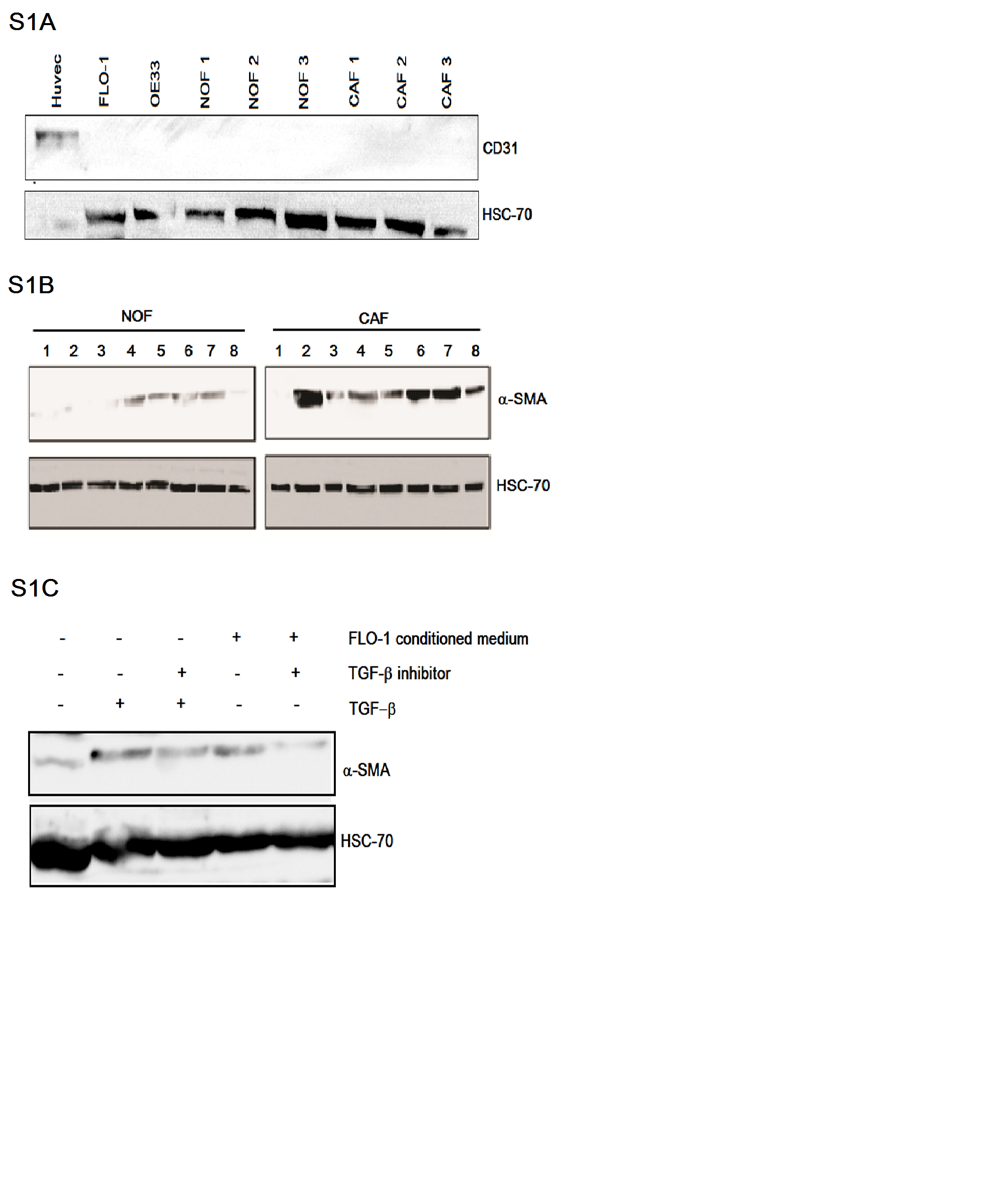

Supplement: Figure S1 — Further characterization of primary oesophageal fibroblasts [file path0235-0466-sd2.tif]

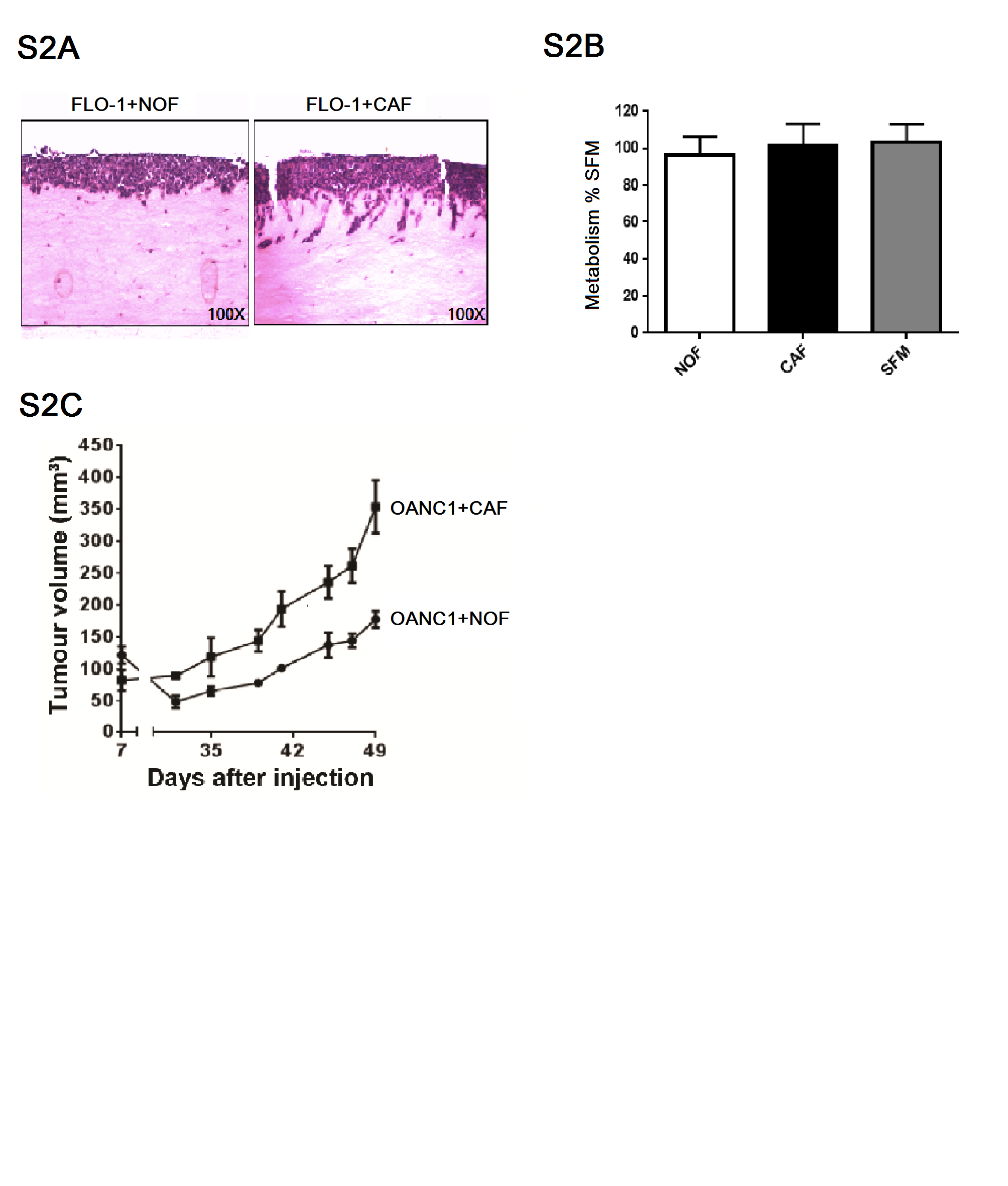

Supplement: Figure S2 — Additional organotypic and mouse xenograft model [file path0235-0466-sd3.tif]

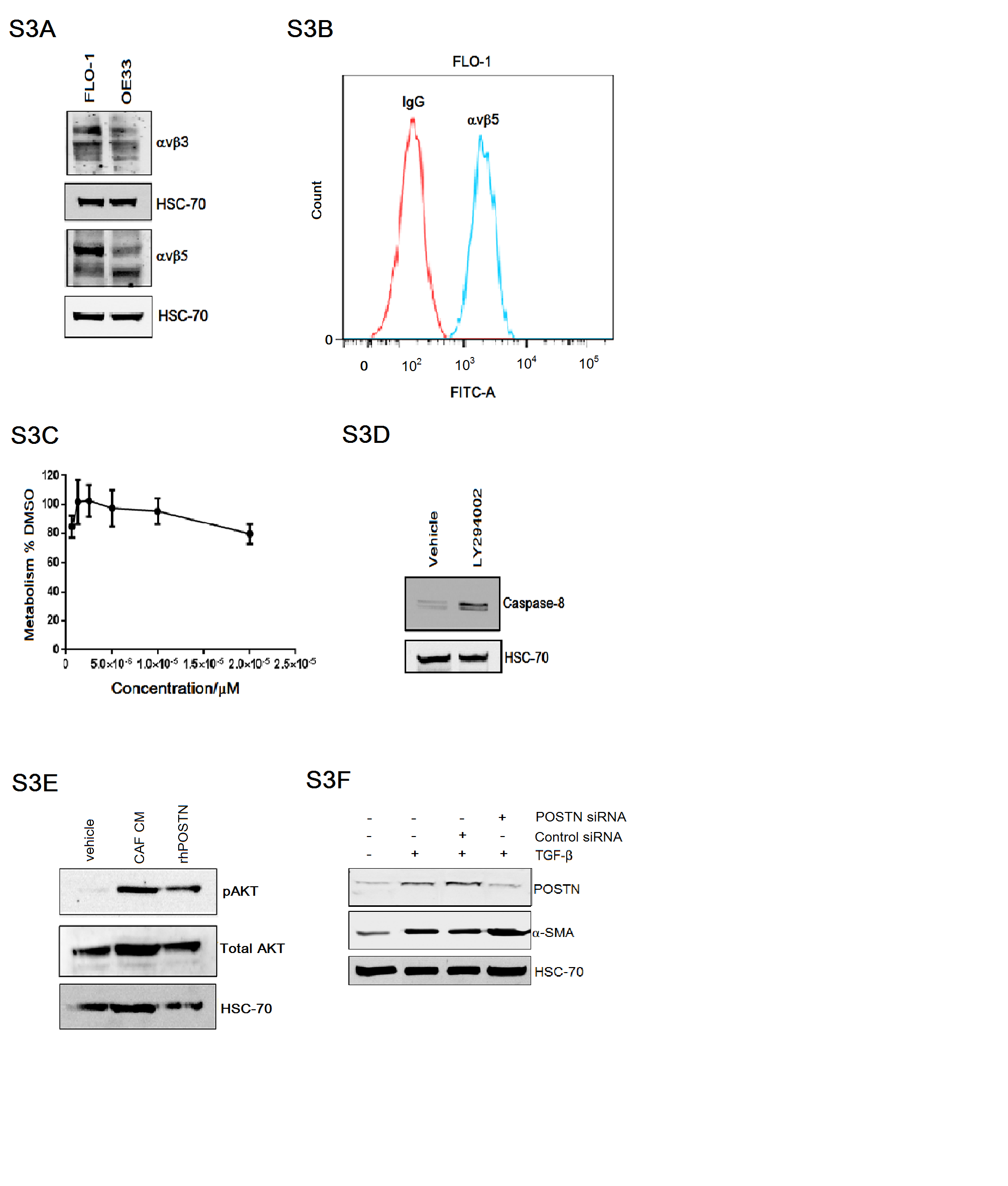

Supplement: Figure S3 — Integrin αvβ3 and αvβ5 expressions were analysed in FLO-1 and OE33 cells using western blot, FACs and MTS proliferation assay [file path0235-0466-sd4.tif]
