# Supplementary material for: Cancer-associated fibroblasts predict poor outcome and promote periostin-dependent invasion in oesophageal adenocarcinoma
Source: J Pathol. 2015 Jan 8;235(3):466–77. doi: 10.1002/path.4467 (PMC4312957; doi:10.1002/path.4467)
Supplement: Table S1 — Clinicopathological characteristics of patient cohort for immunohistochemistry [file path0235-0466-sd5.doc]

|  | | **n=183** |
| --- | --- | --- |
| **Operation Age*** | | 67.61 (36.99-85.41) |
| **Sex ratio (M:F)** | | 155:28 |
| **ASA** | **1** | 16 (8.7) |
|  | **2** | 137 (74.8) |
|  | **3** | 30 (16.5) |
| **Tumour site** | **Lower 1/3** | 61 (33.3) |
|  | **OGJ – S1** | 39 (21.3) |
|  | **OGJ – S2** | 40 (21.9) |
|  | **OGJ – S3** | 43 (23.5) |
| **Type** | **AC** | 183 (100) |
| **pT or ypT** | **T0** | 18 (9.8) |
|  | **T1** | 38 (20.8) |
|  | **T2** | 37 (20.2) |
|  | **T3** | 82 (44.8) |
|  | **T4** | 8 (4.4) |
| **pN or ypN** | **N0** | 93 (50.8) |
|  | **N1** | 39 (21.3) |
|  | **N2** | 29 (15.8) |
|  | **N3** | 22 (12.0) |
| **pM or ypM** | **M0** | 178 (97.3) |
|  | **M1** | 5 (2.3) |
| **Lymph node yield*** | | 17 (3-53) |
| **Resection clearance (R0)** | | 146 (79.8) |
| **Vascular Invasion** | | 63 (34.4) |
| **Lymphatic Invasion** | | 32 (17.5) |
| **Perineural Invasion** | | 24 (13.1) |
| **Neoadjuvant chemotherapy** | | 104 (56.8) |
| Values in parentheses are percentages unless indicated.  *Values in parentheses are range  ASA - American Society of Anesthesiologists physical status classification system | | |

Supplementary Table.1
