# Supplementary material for: Cancer-associated fibroblasts predict poor outcome and promote periostin-dependent invasion in oesophageal adenocarcinoma
Source: J Pathol. 2015 Jan 8;235(3):466–77. doi: 10.1002/path.4467 (PMC4312957; doi:10.1002/path.4467)
Supplement: Table S2 — KME values for all of the proteins clustered in the ECM module [file path0235-0466-sd6.doc]

| **GeneSymbol** | **Ensembl** | **KME ECM** |
| --- | --- | --- |
| POSTN | ENSG00000133110 | 0.87 |
| VCAN | ENSG00000038427 | 0.98 |
| FBN1 | ENSG00000166147 | 0.84 |
| GNG11 | ENSG00000127920 | 0.85 |
| FSTL1 | ENSG00000163430 | 0.9 |
| FERMT2 | ENSG00000073712 | 0.71 |
| FGFR1 | ENSG00000077782 | 0.7 |
| MGP | ENSG00000111341 | 0.82 |
| ZCCHC24 | ENSG00000165424 | 0.81 |
| DPYSL3 | ENSG00000113657 | 0.68 |
| FNDC3B | ENSG00000075420 | 0.84 |
| LHFP | ENSG00000183722 | 0.77 |
| RFTN1 | ENSG00000131378 | 0.91 |
| ITGB1 | ENSG00000150093 | 0.8 |
| COL5A2 | ENSG00000204262 | 0.95 |
| COL3A1 | ENSG00000168542 | 0.91 |
| COL15A1 | ENSG00000204291 | 0.92 |
| PODXL | ENSG00000128567 | 0.95 |
| ANGPTL2 | ENSG00000136859 | 0.9 |
| SDC2 | ENSG00000169439 | 0.84 |
| LAMA4 | ENSG00000112769 | 0.95 |
| COL6A2 | ENSG00000142173 | 0.9 |
| COL6A1 | ENSG00000142156 | 0.88 |
| LGALS1 | ENSG00000100097 | 0.89 |
| ANXA6 | ENSG00000197043 | 0.84 |
| FOXF1 | ENSG00000103241 | 0.85 |
| IGFBP4 | ENSG00000141753 | 0.86 |
| LTBP1 | ENSG00000049323 | 0.91 |
| DPYSL2 | ENSG00000092964 | 0.89 |
| CD93 | ENSG00000125810 | 0.93 |
| SLC39A14 | ENSG00000104635 | 0.78 |
| GPR116 | ENSG00000069122 | 0.91 |
| IGFBP7 | ENSG00000163453 | 0.92 |
| DCHS1 | ENSG00000166341 | 0.87 |
| CTGF | ENSG00000118523 | 0.85 |
| AEBP1 | ENSG00000106624 | 0.84 |
| MMP2 | ENSG00000087245 | 0.87 |
| THBS1 | ENSG00000137801 | 0.93 |
| CYR61 | ENSG00000142871 | 0.91 |
| CRISPLD2 | ENSG00000103196 | 0.9 |
| CD200 | ENSG00000091972 | 0.86 |
| ADAMTS1 | ENSG00000154734 | 0.8 |
| EFEMP2 | ENSG00000172638 | 0.81 |
| PTPRM | ENSG00000173482 | 0.85 |
| RCN1 | ENSG00000049449 | 0.79 |
| ENTPD1 | ENSG00000138185 | 0.87 |
| KDELR2 | ENSG00000136240 | 0.7 |
| LUM | ENSG00000139329 | 0.93 |
| DRAM1 | ENSG00000136048 | 0.82 |
| VPS13C | ENSG00000129003 | 0.64 |
| SPARC | ENSG00000113140 | 0.93 |
| CDR2 | ENSG00000140743 | 0.82 |
| COL4A1 | ENSG00000187498 | 0.95 |
| CHN1 | ENSG00000128656 | 0.87 |
| COL6A3 | ENSG00000163359 | 0.95 |
| CDH5 | ENSG00000179776 | 0.83 |
| PRKCDBP | ENSG00000170955 | 0.83 |
| NR2F2 | ENSG00000185551 | 0.84 |
| LAMC1 | ENSG00000135862 | 0.9 |
| A2M | ENSG00000175899 | 0.94 |
| PXDN | ENSG00000130508 | 0.95 |
| SCCPDH | ENSG00000143653 | 0.69 |
| RARRES2 | ENSG00000106538 | 0.9 |
| TGFBI | ENSG00000120708 | 0.89 |
| MXRA8 | ENSG00000162576 | 0.88 |
| EPB41L2 | ENSG00000079819 | 0.73 |
| SPARCL1 | ENSG00000152583 | 0.67 |
| SERPING1 | ENSG00000149131 | 0.88 |
| COL5A1 | ENSG00000130635 | 0.9 |
| SAFB2 | ENSG00000130254 | 0.63 |
| CDH11 | ENSG00000140937 | 0.92 |
| RRBP1 | ENSG00000125844 | 0.72 |
| STC1 | ENSG00000159167 | 0.86 |
| PRSS23 | ENSG00000150687 | 0.91 |
| TIE1 | ENSG00000066056 | 0.83 |
| EMP3 | ENSG00000142227 | 0.77 |
| SPTBN1 | ENSG00000115306 | 0.7 |
| CBLB | ENSG00000114423 | 0.78 |
| DPY19L1 | ENSG00000173852 | 0.72 |
| NID1 | ENSG00000116962 | 0.92 |
| DAB2 | ENSG00000153071 | 0.91 |
| SEC62 | ENSG00000008952 | 0.67 |
| NUAK1 | ENSG00000074590 | 0.85 |
| MEF2C | ENSG00000081189 | 0.83 |
| COL1A2 | ENSG00000164692 | 0.9 |
| C1S | ENSG00000182326 | 0.95 |
| LOXL2 | ENSG00000134013 | 0.94 |

Supplementary Table 2
